# Repeatability of brain phase-based magnetic resonance electric properties tomography methods and effect of compressed SENSE and RF shimming

**DOI:** 10.1007/s13246-023-01248-1

**Published:** 2023-03-30

**Authors:** Jun Cao, Iain Ball, Peter Humburg, Socrates Dokos, Caroline Rae

**Affiliations:** 1 Neuroscience Research Australia, 139 Barker St, Randwick, NSW 2031 Australia; 2 School of Biomedical Sciences, The University of New South Wales, Kensington, NSW 2052 Australia; 3 Philips Australia & New Zealand, North Ryde, NSW 2113 Australia; 4 Mark Wainwright Analytical Centre, Stats Central, The University of New South Wales, Kensington, NSW 2052 Australia; 5 Graduate School of Biomedical Engineering, The University of New South Wales, Kensington, NSW 2052 Australia; 6 School of Psychology, The University of New South Wales, Kensington, NSW 2052 Australia

**Keywords:** 3 T brain MRI, Brain conductivity, Compressed SENSE, Field mapping, RF shimming, Phase-based magnetic resonance electrical properties tomography

## Abstract

Magnetic resonance electrical properties tomography (MREPT) is an emerging imaging modality to noninvasively measure tissue conductivity and permittivity. Implementation of MREPT in the clinic requires repeatable measurements at a short scan time and an appropriate protocol. The aim of this study was to investigate the repeatability of conductivity measurements using phase-based MREPT and the effects of compressed SENSE (CS), and RF shimming on the precision of conductivity measurements. Conductivity measurements using turbo spin echo (TSE) and three-dimensional balanced fast field echo (bFFE) with CS factors were repeatable. Conductivity measurement using bFFE phase showed smaller mean and variance that those measured by TSE. The conductivity measurements using bFFE showed minimal deviation with CS factors up to 8, with deviation increasing at CS factors > 8. Subcortical structures produced less consistent measurements than cortical parcellations at higher CS factors. RF shimming using full slice coverage 2D dual refocusing echo acquisition mode (DREAM) and full coverage 3D dual TR approaches further improved measurement precision. BFFE is a more optimal sequence than TSE for phase-based MREPT in brain. Depending on the area of the brain being measured, the scan can be safely accelerated with compressed SENSE without sacrifice of precision, offering the potential to employ MREPT in clinical research and applications. RF shimming with better field mapping further improves precision of the conductivity measures.

## Introduction

Phase-based MREPT using the ${B}_{1}$ phase map may be particularly useful for clinical applications [1, 2, 3, 4, 5, 6, 7, 8], and, to this end, there have been a variety of approaches to the implementation of MREPT [9, 10, 11, 12, 13, 14, 15, 16]. Conductivity is a potential biomarker of tissue physiology, especially pathophysiology, to provide additional contrast between normal and abnormal tissues. Elevated conductivity compared to normal tissues was reported in tumors in the brain [1], breast [2, 3] and pelvis [4, 5] as well as in the ischemic infarction area caused by an ischemic brain stroke [6]. Phase-based MREPT is able to differentiate benign and malignant tumors [7, 8]. A breast cancer study of 90 subjects revealed that malignant cases showed increased conductivity compared to benign cases, and that invasive cancers showed higher mean conductivity than cancers in situ [7]. Another study involving 116 patients with breast lesions found the diagnostic ability of phase-based MREPT to differentiate between benign and malignant breast lesions was comparable to that of standard dynamic contrast-enhanced MRI (DCE-MRI) with the added advantage that no contrast medium was required for phase-based MREPT [8]. While further studies are needed to better define the potential of MREPT as a clinical diagnostic, the evidence in support of its use is so far compelling.

Phase-based MREPT is based on the transceive phase assumption that the phase of the positively-rotating component of ${B}_{1}$ is half of the image’s transceive phase. In principle, the performance of phase-based MREPT depends on the phase image quality. In a study of phase-based MREPT at 1.5 T, 3 T and 7 T, the transceive phase assumption was found to be applicable at magnetic field strengths of 1.5 T and 3 T [17]. Transceive phase is prone to unwanted phase contributions from off-resonance effects and eddy currents. Sequences based on refocusing pulses, such as spin echo (SE) [18, 19] and turbo spin echo (TSE) [20], are noted for exclusion of off-resonance effects. In the first successful MREPT experiment, spin echo was used to derive the conductivity map of the heart and liver of a dog [21]. Another sequence frequently used for MREPT is balanced fast field echo (bFFE) [22, 23]. bFFE has a high signal-to-noise ratio (SNR) with a short acquisition time, and the SNR of bFFE is dependent on pulse sequence efficiency [24], which implies that maximum available gradient strength and slew rates can be used to improve the SNR. The off-resonance effects in bFFE are negligible and the transceive phase of bFFE was shown to resemble that of SE [25]. bFFE suffers from banding artifacts, which often occur at locations where the main field inhomogeneity is a multiple of 1/TR. Short-TR bFFE could greatly reduce banding artifacts and preserve the quality of its phase image.

In principle, the performance of phase-based MREPT depends on the phase image quality. Apart from denoising filters, convolutional neural networks have been tried to denoise the phase maps [26]. Deep neural networks, from conditional generative adversarial network [27], convolutional neural networks [28, 29] to physics informed neural networks [30], have also been attempted to reconstruct conductivity from phase based on electromagnetic simulations. The recently proposed physics informed neural networks showed robust improvement to conductivity maps extracted from noisy images in numerical simulations, and showed feasibility in a phantom study, although the influence of noise and boundary artefacts still remained [30].

Implementation of MREPT in the clinic requires a robust and repeatable method as well as a short scan time. This could be accomplished using compressed SENSE [31] but the effect of this on the precision and repeatability of the calculated variables is unknown. Here, we investigated the repeatability of two different MREPT sequences, TSE and bFFE, and the impact of compressed SENSE on the repeatability of bFFE with compressed SENSE factors from 1.3 to 12.

As phase-based MREPT derives conductivity from the phase map while assuming that the magnitude of ${B}_{1}^{+}$ is constant in the region of interest, the uniformity of ${B}_{1}^{+}$ affects the error of MRI measurement and the precision of corresponding conductivity reconstruction. Parallel RF transmission using the multi-channel body coil results in a more uniform ${B}_{1}$ field [32]. RF shimming comprises: ${B}_{1}^{+}$ field mapping for each transmit coil, and selection of the optimized operating parameters for amplitude and phase to meet a uniformity criterion. Since a ${B}_{1}$ map of each channel of the body coil is acquired for RF shimming to determine the optimal ${B}_{1}^{+}$ magnitude and phase settings for each channel to realize the most uniform transmit field, we are interested in the effect of ${B}_{1}^{+}$ mapping using different techniques.

As $\left|{B}_{1}^{+}\right|$ is proportional to flip angle $\alpha$ if pulse duration time is short, the problem of mapping $\left|{B}_{1}^{+}\right|$ for RF shimming can be turned into mapping the flip angle distribution. The dual-TR method (actual flip angle imaging, AFI) uses a sequence consisting of interleaved scans of flip angles with two different TRs [33]. The flip angle can be derived by the ratio of image intensities and the ratio of TRs.

Dual refocusing echo acquisition mode (DREAM) uses a stimulated echo acquisition mode preparation sequence (STEAM), and a single-shot, low-angle, gradient echo sequence to read out the prepared magnetization [34]. The stimulated signal and free induction decay (FID) signal are acquired at the same time, and the ratio of the two signals is used to derive the flip angle of the STEAM preparation pulses.

Here, we compared the effect of three ${B}_{1}^{+}$ mapping methods for RF shimming, 3D dual TR, single-slice 2D DREAM and full slice coverage 2D DREAM on phase-based MREPT. This work was essential to underpin the investigations undertaken to establish the precision of the measurement and hence the reliability of the data we obtained.

The aims of the work were:

To determine which of the commonly used sequences for MREPT, bFFE or TSE gave the more precise measurements, and;

To determine which field mapping approach calibrated for RF shimming provides the best measurement precision in the subsequent tissue conductivity mapping, and;

To determine how much acceleration could be applied to the bFFE sequence without significant penalty and loss of precision.

## Material and methods

### Magnetic resonance acquisition

All scans in this study were acquired at 3 T (Ingenia CX, Philips Healthcare, Best, The Netherlands) using a 32-channel head coil.

#### Measurement of conductivity in known saline solutions using bFFE

Two independent cylindrical compartments were filled with different saline solutions, 3.3 g/L and 6.6 g/L respectively, with conductivity values 0.540 S/m and 1.069 S/m at the measured temperature 21.5 °C [35]. The phantom underwent T1W TFE (turbo field echo), and two repeated bFFE scans with compressed SENSE factor 1.

Parameters for T1W TFE were: TR/TE = 7.46/3.47 ms, resolution 1 $\times$ 1 $\times$ 1 ${\mathrm{mm}}^{3}$, sagittal slices, FOV 192 $\times$ 150 ${\mathrm{mm}}^{2}$, flip angle 8$^\circ$. Parameters for bFFE were: TR/TE = 2.54/1.27 ms, resolution 1 $\times$ 1 $\times$ 1 ${\mathrm{mm}}^{3}$, sagittal slices, FOV 240 $\times$ 140 ${\mathrm{mm}}^{2}$, nonselective RF pulses, flip angle 25$^\circ$, compressed SENSE factor 1, RF shimming calibrated with full-coverage 2D DREAM.

#### Repeatability test of TSE and bFFE with compressed SENSE

Both bFFE and TSE sequences were tested to determine the better option for phase-based MREPT. In addition, we acquired bFFE scans with increasing compressed SENSE factors to determine the impact of accelerated scan time on precision.

Five healthy subjects (four females, one male, age 32.2 $\pm$ 12.8 years) were scanned with the scan repeated on a separate day at a similar time of day. For each session, the subject underwent T1W TFE (turbo field echo), TSE and bFFE scans with compressed SENSE factors from 1.3 to 12. To minimize the effect of scan order, TSE and bFFE scan order was randomized for each session of each subject.

Parameters for T1W TFE were: TR/TE = 5.89/2.76 ms, resolution 1 $\times$ 1 $\times$ 1 ${\mathrm{mm}}^{3}$, sagittal slices, FOV 240 $\times$ 240 ${\mathrm{mm}}^{2}$, flip angle 8$^\circ$. Parameters for TSE were: TR/TE = 3000/250 ms, resolution 1 $\times$ 1 $\times$ 1 ${\mathrm{mm}}^{3}$, sagittal slices, FOV 240 $\times$ 240 ${\mathrm{mm}}^{2}$. Parameters for bFFE were: TR/TE = 2.45/1.23 ms, resolution 1 $\times$ 1 $\times$ 1 ${\mathrm{mm}}^{3}$, sagittal slices, FOV 240 $\times$ 240 ${\mathrm{mm}}^{2}$, nonselective RF pulses, flip angle 25 $^\circ$, compressed SENSE factors 1.3/4/6/8/10/12. The scan durations of bFFE were 3 min 31 s, 1 min 12 s, 48 s, 36 s, 28 s, and 24 s for compressed SENSE factors 1.3, 4, 6, 8, 10 and 12 respectively.

Additionally, a fast acquisition (dynamic scan time 1.89 s) was tested by scanning one healthy subject (age: 30, male) using bFFE with CS factor 4. Parameters for bFFE were: TR/TE = 1.58/0.79 ms, nonselective RF pulses, flip angle 25$^\circ$, resolution 3 $\times$ 3 $\times$ 3 ${\mathrm{mm}}^{3}$, sagittal slices, 520 dynamics.

#### RF shimming calibrated with ${B}_{1}$ mapping

The coil sensitivity profiles for the receive elements of the head coil are determined by normalization with the reference body coil, through a SENSE reference prescan, which acquires an image from each receive element of the surface head coil, as well as an image from the body coil serving as the reference coil when using a vendor-specific algorithm, Constant Level Appearance—CLEAR for homogeneity correction [36]. The received uniformity of the body coil in the homogeneity sphere is mainly determined by the anatomy and the RF shimming capabilities. The Ingenia CX scanner used here is a two-channel MultiTransmit system, where the ${B}_{1}^{+}$ magnitude and phase of each channel is altered in a subject-adaptive way, shimming the ${B}_{1}^{+}$ to the subject’s anatomy. By RF shimming the ${B}_{1}^{+}$ using MultiTransmit, it ends up shimming the receive field ${B}_{1}^{-}$ through the principle of reciprocity. Then, the body coil can be used as homogeneous reference for CLEAR to correct image intensity variation [37]. Since a ${B}_{1}$ map of each channel of the body coil is acquired for RF shimming to determine the optimal ${B}_{1}^{+}$ magnitude and phase settings for each channel to realize the most uniform transmit field, ${B}_{1}^{+}$ mapping methods might have an effect on RF shimming.

Comparisons of ${B}_{1}$ mapping methods were tested by scanning four healthy subjects (age: 40.3 $\pm$ 11.1, one female, three males) at 3 T. Two bFFE scans were acquired for each ${B}_{1}$ mapping method, including single-slice 2D DREAM, full slice coverage 2D DREAM and full slice coverage 3D dual TR. Parameters for bFFE were: TR/TE = 2.5/1.25 ms, nonselective RF pulses, flip angle 25 $^\circ$, sagittal slices, FOV 240 $\times$ 240 ${\mathrm{mm}}^{2}$, acquired resolution 1.3 $\times$ 1.3 $\times$ 1 ${\mathrm{mm}}^{3}$ reconstructed to 1 $\times$ 1 $\times$ 1 ${\mathrm{mm}}^{3}$.

A single-slice 2D DREAM was the default ${B}_{1}$ mapping for RF shimming in this study, acquired axially with a much larger FOV than that of bFFE. The full slice coverage 3D dual TR and full slice coverage 2D DREAM were acquired sagittally with the same FOV as that of bFFE. In the full slice coverage 3D dual TR approach, the number of sagittal slices was extended to cover the right ear to the left ear. The full slice coverage 2D DREAM was accomplished through multiple 2D slices with some slice gap in between to achieve a whole brain coverage.

Parameters for single slice 2D DREAM acquisition were: one axial slice, FOV 530 $\times$ 470 ${\mathrm{mm}}^{2}$, imaging slice thickness 20 mm, non-selective STEAM flip angle 120$^\circ$, imaging flip angle 15$^\circ$, TE_STE_ = 1.35 ms, TE_FID_ = 2.3 ms, TR = 3.9 ms, scan duration 1.9 s.

Parameters for full slice coverage 2D DREAM acquisition were: 12 sagittal slices, FOV 240 $\times$ 240 ${\mathrm{mm}}^{2}$, imaging slice thickness 15 mm, slice gap 1.5 mm, non-selective STEAM flip angle 60$^\circ$, imaging flip angle 15$^\circ$, TE_STE_ = 1.47 ms, TE_FID_ = 2.3 ms, TR = 4.2 ms, scan duration 37.1 s.

Parameters for full slice coverage 3D dual TR acquisition were: 24 sagittal slices, 240 $\times$ 240 ${\mathrm{mm}}^{2}$, imaging slice thickness 8 mm, non-selective, imaging flip angle 60°, TE = 1.48 ms, TR_1_ = 20 ms, TR_2_ = 100 ms, scan duration 144.7 s.

Bland–Altman plot analysis was used to compare the repeatability of conductivity values reconstructed from bFFE using different ${B}_{1}^{+}$ mapping methods.

### Conductivity reconstruction

As the measured transceive phase was wrapped into the range between 0 and $2\pi$ radians, phase unwrapping is required before using phase for conductivity calculation. The phase unwrapping method used in this paper was Speedy rEgion-Growing Algorithm for Unwrapping Estimated Phase (SEGUE) [38]. SEGUE divides the $[\mathrm{0,2}\pi )$ phase interval into six smaller intervals, determines connected 3D regions, and gradually enlarges the region with the largest border by unwrapping and merging the adjacent regions. SEGUE can provide similarly accurate results as the gold standard method, Phase Region Expanding Labeller for Unwrapping Discrete Estimates (PRELUDE) [39], and works 1.5 to 70 times faster than PRELUDE depending on echo time and anatomical regions, which makes SEGUE suitable for high-resolution phase unwrapping for MREPT.

Denoising is often implemented for phase-based MREPT because the Laplacian operation is sensitive to noise. Linear filters were found to either underestimate or overestimate the conductivity and permittivity values, while anisotropic diffusion filters performed better at the cost of computation time [40]. An adaptive nonlinear denoising filter was proposed to improve the image quality of reconstructed electrical properties at a reduced computation time [40], which uses parameters from a geometric model to differentiate noisy voxels from voxels on the edges in the three dimensions. This data-driven denoising filter is applicable to real and imaginary parts of the complex ${B}_{1}$ field, or its magnitude and phase, which will improve the conductivity and permittivity images to different degrees. In our experiment, the adaptive nonlinear filter was applied to the unwrapped phase to benefit more conductivity reconstruction, with number of iterations 400, constant integration 0.18, and Sigmoid diffusivity function.

After phase unwrapping and denoising, calculation of phase-based MREPT conductivity was performed voxel-wise. In this study, phase-based MREPT was adopted as expressed below:

$$\sigma \approx \frac{{\nabla^{2} \varphi_{ \pm }^{ } }}{{2\mu_{0 } \omega }}$$

where $\sigma$ denotes conductivity (unit: S/m), ${\mu }_{0}=4\pi \times {10}^{-7}$ H/m is the magnetic permeability of free space, $\omega$ denotes the Larmor angular frequency, ${\nabla }^{2}$ denotes the Laplacian operation, and ${\varphi }_{\pm }$ denotes the transceive phase.

Numerical calculation of the Laplacian involves a voxel ensemble/kernel around the target voxel. Voxels near tissue boundaries have neighboring voxels belonging to difference tissue types. In this study, T1W TFE scans were acquired and co-registered for segmenting brain into three main tissue types, white matter (WM), gray matter (GM) and cerebrospinal fluid (CSF) by FSL (FMRIB, Oxford, UK) [41], to help alleviate boundary artifacts in the conductivity calculation.

Within each tissue type, an average parabolic phase fitting method was used to reduce artifacts [2], which were amplified by the Laplacian operation of phase data, especially when expressed in three-dimensional Cartesian coordinates. Taking the horizontal dimension of a 2D phase image for example, parabolic fitting could be conducted with the target voxel and the voxels on its left or right side, leading to two quadratic expressions of phase around the target voxel. The second derivative of the target phase was assigned by the average of second derivatives of the two quadratic expressions. Similarly, second derivatives of the target phase in another two dimensions can be calculated, to obtain the Laplacian. The kernel was limited to voxels which had close image amplitudes to the target voxel, to further reduce boundary artifacts. The kernel size had an effect on the noise in conductivity reconstruction [42] and the maximum kernel size of voxels was set as 9 × 9 × 6. And for each target voxel, the fitted phase was only accepted if the correlation coefficient between fitted phase and measured phase was larger than 70%. After the Laplacian of the phase image was calculated, the conductivity image was obtained with angular frequency $\omega =2\pi \bullet 127.76\times {10}^{6}$ rad/s for the 3 T MRI scanner used in this study.

### Statistical analysis

Here, we aimed to examine the repeatability of brain conductivity measurement by phase-based MREPT using TSE, bFFE with compressed SENSE factors 1.3 up to 12 respectively, and compare the performance of these sequences. Firstly, a two-sample equivalence test was used to determine if conductivity measurements of 22 anatomical ROIs using different sequences were equivalent. The anatomical ROIs were subcortical structures and cortical divisions based on brain parcellations of T1W TFE images using the Freesurfer image analysis suite (http://surfer.nmr.mgh.harvard.edu/) [43], including putamen, caudate, thalamus, hippocampus, white matter, amygdala, frontal lobe, parietal lobe, temporal lobe, precentral lobe, postcentral lobe and lateral occipital lobe. Secondly, ANOVA tests were applied to evaluate if there was a significant difference in brain conductivities between the two samples. ANOVA was applied to both repeated sessions of the same sequence and sessions of two different sequences. Significance level was taken as 0.05 for the ANOVA and two-sample equivalence test. Thirdly, Bland–Altman plots were produced based on the 22 anatomical ROIs. Mean of difference (bias) and limits of agreement (LoA: mean ± 1.96 SD) were used to compare the difference of conductivity measurements using different sequences.

To examine the effects of RF shimming calibrated with different field mapping methods, Bland–Altman plots were plotted based on the mean conductivity values and standard deviations of gray matter, white matter and CSF of reconstructed conductivity maps from bFFE using different field mapping approaches. Paired t-tests were conducted to compare the difference between conductivity measurements from these scans.

## Results

### Phantom test using bFFE

The conductivity measurements of the two cylindrical compartments from the two repeated bFFE scans were 0.534 ± 0.019 S/m, 0.536 ± 0.022 S/m (true value: 0.540 S/m) and 1.048 ± 0.065 S/m, 1.053 ± 0.071 S/m (true value: 1.069 S/m), with coefficient of variation 3.56%, 4.10% and 6.20%, 6.74%, respectively.

### Comparisons of reconstructed conductivity using bFFE and TSE

**Fig. 1:**
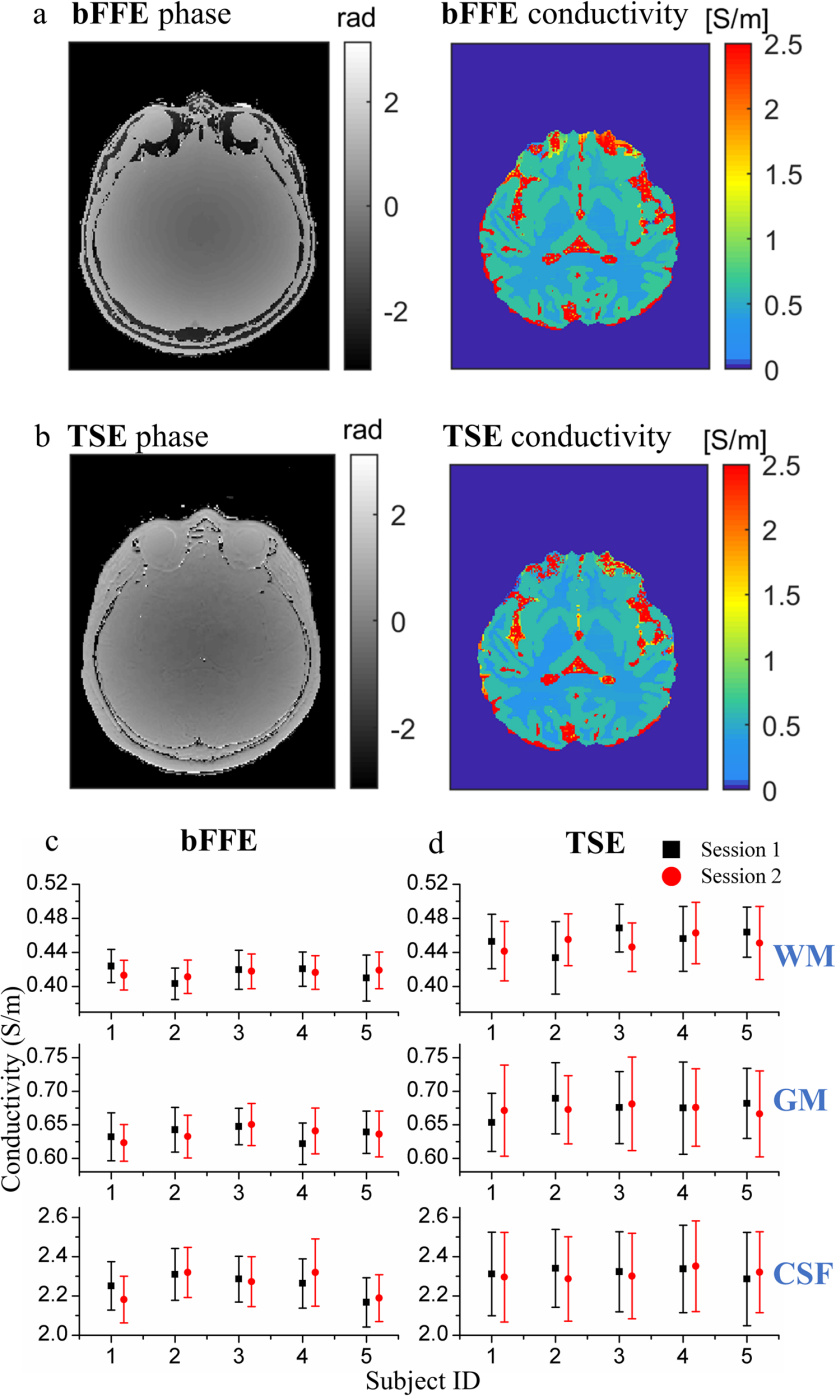
Conductivity measurements from five healthy subjects undergoing scans at similar times on two different days. **a** Phase and conductivity images of one axial slice using bFFE. **b** Phase and conductivity images of the same axial slice using TSE. **c** Conductivity values (with standard deviations) of WM, GM and CSF from five subjects, measured by bFFE on two different days. **d** Conductivity values (with standard deviations) of WM, GM and CSF from five subjects, measured by TSE on two different days

Figure 1a and b show phase and conductivity images of the same axial slice measured from bFFE and TSE sequences, respectively. TSE can provide a more complete phase image of the whole head than bFFE. The conductivity map of brain tissues from bFFE overall resembles that from TSE. Figure 1c and d show conductivity values (with standard deviations) of white matter, gray matter and CSF of five healthy subjects on two sessions, measured from bFFE and TSE respectively. The mean and variance of conductivity measurement of TSE were larger than those of bFFE. The conductivity values measured using bFFE were 0.42 $\pm$ 0.02 S/m for white matter (coefficient of variation 4.76%), 0.64 $\pm$ 0.03 S/m for gray matter (coefficient of variation 4.69%), and 2.24 $\pm$ 0.13 S/m for CSF (coefficient of variation 5.8%). The conductivity values measured using TSE were 0.45 $\pm$ 0.03 S/m for white matter (coefficient of variation 6.67%), 0.67 $\pm$ 0.06 S/m for gray matter (coefficient of variation 8.96%), and 2.32 $\pm$ 0.22 S/m for CSF (coefficient of variation 9.48%). Table 1 shows the conductivity measurements of brain tissues at 3 T from healthy human participants in the literature, including an ex vivo probe measurement study [44, 45], and three in vivo MREPT studies [10, 46, 47]. Phase-based MREPT implemented in this paper provides one of the more precise measurements of brain tissue conductivity reported so far.

**Table 1:** Conductivity measurements of brain tissues from healthy human participants at 3 T

Literature	Conductivity of brain tissues (S/m; Mean ± SD)		
	GM	WM	CSF
This paper (In vivo)	0.64 ± 0.03	0.42 ± 0.02	2.24 ± 0.13
Gabriel et al., 1996 [44, 45] (Ex vivo probe)	0.59	0.34	2.14
Voigt et al., 2011 [10] (In vivo)	0.72 ± 0.15	0.43 ± 0.15	1.82 ± 0.37
Michel et al., 2017 [46] (In vivo)	0.634 ± 0.06	0.347 ± 0.01	2.225 ± 0.16
Liao et al., 2019 [47] (In vivo)	0.67 ± 0.2	0.51 ± 0.1	

### Repeatability and comparisons of reconstructed conductivity using bFFE with increasing CS factors

**Fig. 2:**
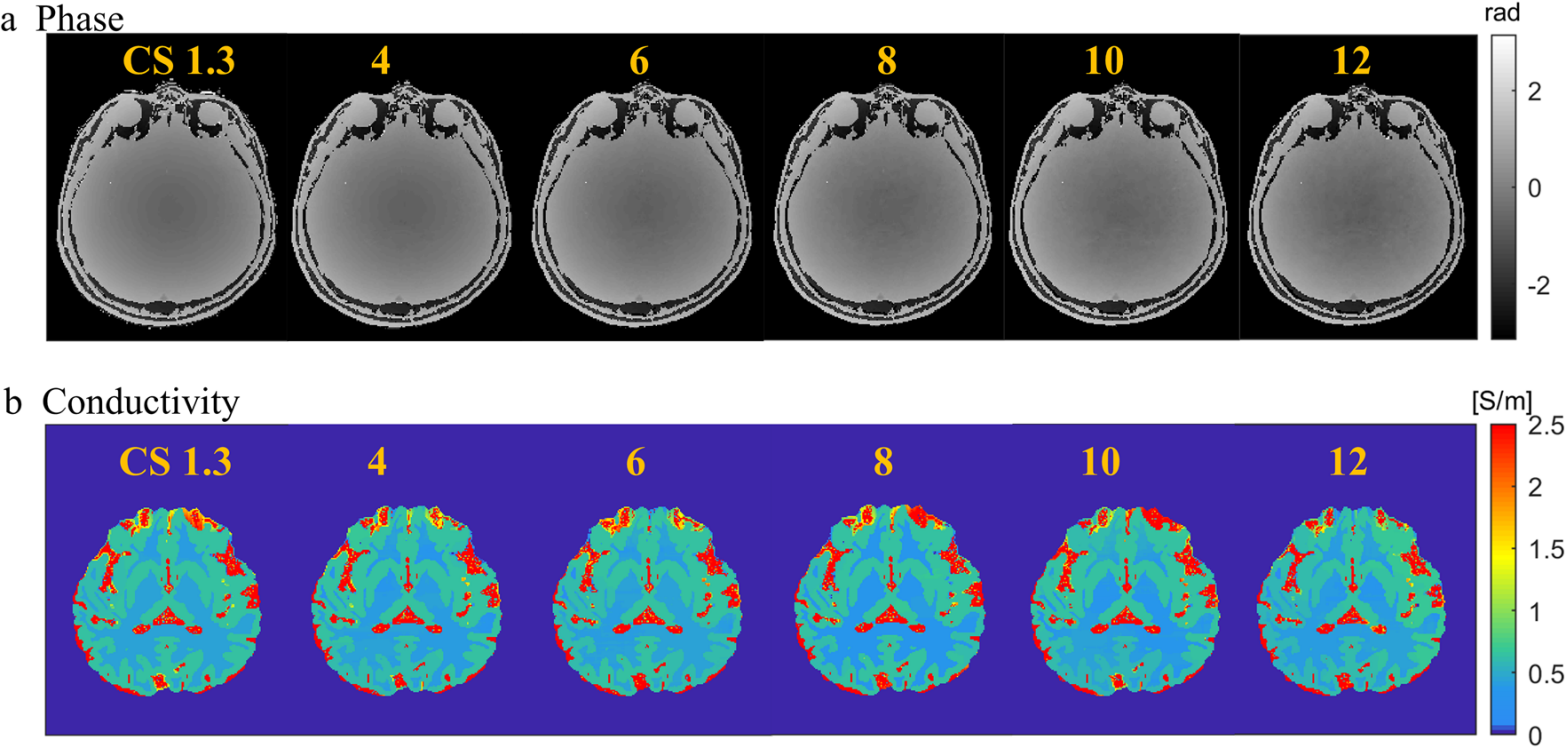
Conductivity maps obtained using bFFE with increasing compressed SENSE factors. **a** Phase images of the same axial slice using bFFE with compressed SENSE factors increasing from 1.3 to 12. **b** Conductivity images of the same axial slice using bFFE with compressed SENSE factors increasing from 1.3 to 12

**Fig. 3:**
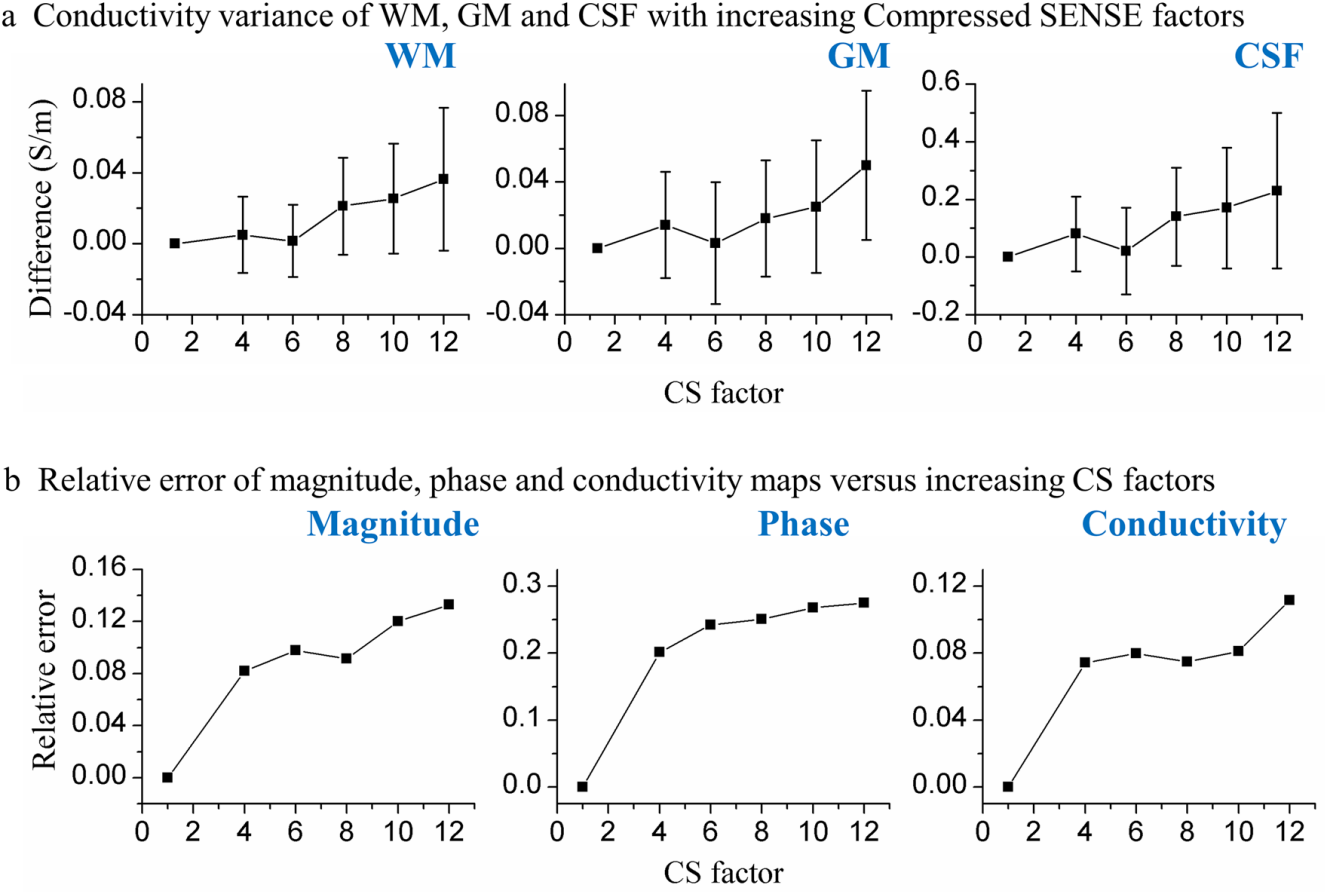
Conductivity variance and relative error by bFFE with increasing compressed SENSE factors. **a** Conductivity variance of WM, GM and CSF with increasing compressed SENSE factors from 1.3 to 12 using CS 1.3 as reference. **b** Relative error of magnitude, phase and conductivity maps with compressed SENSE factors increasing from 1.3 to 12

Figure 2a and b show phase and conductivity images of bFFE sequences with compressed SENSE factors increasing from 1.3 to 12. Compressed SENSE factor 1.3 was used as the reference scan. There was no obvious change in the phase and conductivity images with CS factors up to 8. Figure 3a shows the conductivity variation of white matter, gray matter and CSF with increasing CS factors. Conductivity difference was calculated by subtracting conductivity maps with higher CS factors (from 4 to 12) from the reference conductivity map (CS factor 1.3). The conductivity measurement showed minimal deviation with CS factors up to 8 with deviations increasing at CS factors > 8.

Relative error can be used to quantify the difference between two images ${I}_{1}$,${I}_{2}$ using the ${l}_{2}$ norm [48], defined as

$$\frac{{\Vert {I}_{1}-{I}_{2}\Vert }_{{l}_{2}}}{{\Vert {I}_{1}\Vert }_{{l}_{2}}}$$

Figure 3b shows the relative error of magnitude, phase and conductivity maps with increasing CS factors. bFFE CS4 to CS8 had smaller errors relative to bFFE CS1.3.

To test to what extent different parts of the brain will continue to provide reliable measurements with increasing compressed SENSE factors, we used two-sample equivalence tests of the absolute differences to measurements obtained at CS 1.3. The corresponding *p*-values and upper bounds of confidence intervals of 22 anatomical ROIs are listed in Table 2 (the comparisons between bFFE CS1.3 and other sequences). The null hypothesis was that the absolute difference between the means of the two conductivity measurements exceeded 0.025 S/m. The equivalence margin was set as 0.025 S/m, the same as the standard deviation of WM conductivity measurement using bFFE CS1.3. The statistical power of the equivalence test varied with different CS factors. In the worst scenario where bFFE CS12 had the largest standard deviation 0.045 S/m, the statistical power was 0.866 when equivalence margin = 0.025 S/m, sample size = 5 and correlation coefficient = 0.95. *P-*values less than 0.05 indicated that the two measurements were equivalent. Taking the conductivity measurement using bFFE CS1.3 as reference, with CS factors increasing up to 12, subcortical structures typically had conductivity measurement nonequivalent to the reference, while most cortical sub-divisions had conductivity measurement equivalent to the reference.

**Table 2:** Repeatability of conductivity measures across sub-cortical brain regions using different acquisition methods

Structure	Brain part	Method 1	CS1.3					
		Method 2	TSE	CS4	CS6	CS8	CS10	CS12
Putamen	Left		**0.130 [0.048]**	0.003 [0.024]	0.013 [0.016]	0.006 [0.021]	**0.118 [0.029]**	**0.231 [0.034]**
	Right		**0.223 [0.037]**	0.005 [0.013]	0.016 [0.024]	0.001 [0.020]	0.031 [0.023]	**0.225 [0.028]**
Caudate	Left		**0.155 [0.029]**	0.023 [0.024]	0.009 [0.012]	**0.479 [0.044]**	**0.101 [0.032]**	**0.729 [0.037]**
	Right		**0.142 [0.027]**	0.002 [0.024]	0.003 [0.016]	**0.415 [0.035]**	**0.279 [0.032]**	**0.168 [0.037]**
Thalamus	Left		**0.195 [0.032]**	0.002 [0.020]	0.036 [0.019]	0.001 [0.019]	0.012 [0.023]	**0.076 [0.029]**
	Right		**0.228 [0.035]**	0.011 [0.021]	0.021 [0.016]	0.037 [0.024]	**0.053 [0.026]**	**0.101 [0.031]**
Hippocampus	Left		0.036 [0.022]	0.041 [0.019]	**0.112 [0.033]**	**0.423 [0.036]**	0.042 [0.022]	**0.285 [0.042]**
	Right		**0.228 [0.031]**	**0.165 [0.035]**	0.035 [0.018]	0.011 [0.020]	**0.241 [0.028]**	**0.241 [0.042]**
Amygdala			0.020 [0.016]	0.013 [0.014]	0.011 [0.015]	**0.322 [0.037]**	**0.187 [0.027]**	**0.187 [0.041]**
White matter	Left		0.011 [0.019]	0.007 [0.017]	0.090 [0.013]	0.001 [0.016]	0.016 [0.017]	0.023 [0.019]
	Right		0.016 [0.018]	0.021 [0.019]	0.037 [0.015]	0.033 [0.024]	0.045 [0.019]	**0.098 [0.026]**
Frontal lobe	Superior frontal		0.011 [0.020]	0.015 [0.009]	0.004 [0.015]	**0.148 [0.029]**	**0.416 [0.034]**	**0.337 [0.045]**
	Caudal middle frontal		**0.137 [0.032]**	**0.121 [0.028]**	0.002 [0.017]	**0.322 [0.037]**	**0.566 [0.042]**	**0.597 [0.043]**
	Rostral middle frontal		0.028 [0.021]	0.019 [0.016]	0.002 [0.015]	0.015 [0.017]	0.014 [0.019]	0.013 [0.019]
Parietal lobe	Superior parietal		**0.064 [0.029]**	0.024 [0.019]	0.033 [0.020]	**0.062 [0.026]**	0.009 [0.012]	0.025 [0.011]
	Inferior parietal		0.011 [0.017]	0.002 [0.018]	0.012 [0.013]	0.042[0.020]	**0.063 [0.036]**	**0.136 [0.047]**
Temporal lobe	Superior temporal		**0.193 [0.035]**	0.015 [0.012]	0.047 [0.014]	**0.073 [0.026]**	**0.264 [0.031]**	**0.130 [0.042]**
	Middle temporal		0.009 [0.019]	0.003 [0.013]	0.004 [0.020]	**0.248 [0.042]**	**0.195 [0.033]**	**0.162 [0.040]**
	Inferior temporal		0.018 [0.021]	0.001 [0.016]	0.010 [0.016]	**0.106[0.029]**	0.003 [0.032]	0.032 [0.018]
Precentral lobe			0.011 [0.017]	0.001 [0.013]	0.032 [0.014]	0.012 [0.018]	0.027 [0.023]	0.034 [0.024]
Postcentral lobe			0.044 [0.022]	0.017 [0.010]	0.016 [0.014]	0.004 [0.016]	0.026 [0.014]	**0.087 [0.026]**
Lateral occipital lobe			**0.607 [0.039]**	0.038 [0.017]	0.032 [0.024]	0.020 [0.022]	0.038 [0.020]	**0.151 [0.028]**

*p-values (two-sample equivalence test) and [the absolutely larger bound of confidence intervals of conductivity difference]. Values in bold are not statistically equivalent to Method 1 (CS 1.3). (α = 0.05)

The lack of significance for some of the equivalence tests could be due to bias introduced by the acquisition method or be an indication of insufficient power due to increased variability of the measurements. ANOVA tests were used to investigate to what extent bias can explain the observed differences. The corresponding *p*-values are listed in Table 3, and were considered significant where *p* ≤ 0.05. The conductivity measurements of two sessions were generally repeatable, using the same sequence (TSE, bFFE CS1.3 up to 12). The conductivity measurements using bFFE CS1.3 were significantly different to those measured with TSE as well as, bFFE CS10 and CS12.

**Table 3:** Repeatability and comparison of sequences

Method 1	Method 2	P value
TSE	TSE	0.589
bFFE CS1.3	bFFE CS1.3	0.632
bFFE CS4	bFFE CS4	0.647
bFFE CS6	bFFE CS6	0.412
bFFE CS8	bFFE CS8	0.377
bFFE CS10	bFFE CS10	0.193
bFFE CS12	bFFE CS12	0.074
bFFE CS1.3	TSE	0.039*
	bFFE CS4	0.243
	bFFE CS6	0.112
	bFFE CS8	0.265
	bFFE CS10	0.043*
	bFFE CS12	0.032*

*p*-values derived from ANOVA

^*^(α = 0.05, Bonferroni correction)

Taken together the results show that bFFE at CS factors up to 8 is highly repeatable. TSE is repeatable, with slightly larger errors than CS 1.3 bFFE, but it yields significantly higher conductivity values.

**Fig. 4:**
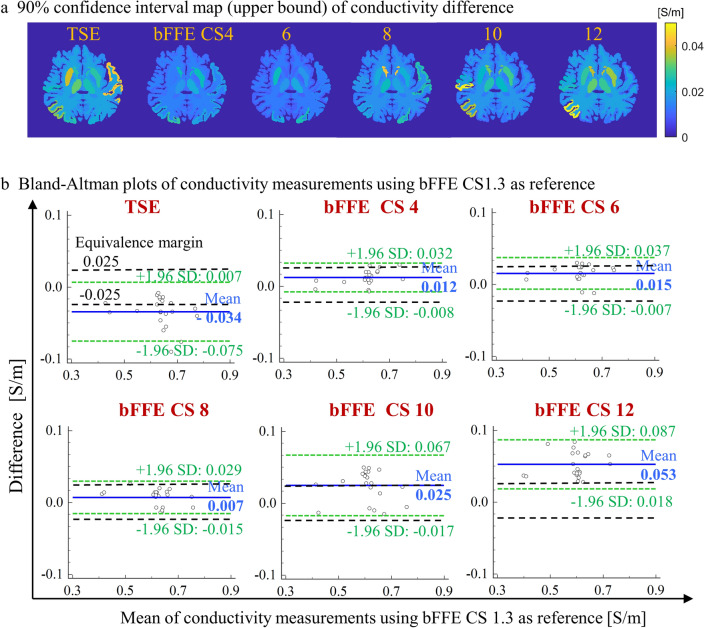
Conductivity measurement by bFFE with increasing compressed SENSE factors. **a** Upper bound of the 90% confidence interval map of the absolute conductivity difference between bFFE CS1.3 and other sequences (TSE minus bFFE CS1.3, bFFE CS1.3 minus bFFE with higher CS factors). **b** Bland–Altman plots of conductivity measurements between bFFE CS 1.3 and other sequences, bFFE CS1.3 minus other sequences (Mean: mean of difference; LoA: limits of agreement, mean ± 1.96 SD; equivalence margin: ± 0.025 for the equivalence test)

TSE showed conductivity measurement nonequivalent to the reference both in subcortical structures and cortical divisions. Figure 4a visualizes the upper bound of the 90% confidence intervals of the absolute conductivity difference between bFFE CS1.3 and other sequences, which also indicates that with increasing CS factors, more subcortical structures and cortical divisions had a larger confidence interval of conductivity difference with the reference.

Figure 4b shows Bland–Altman plots of conductivity measurements of 22 anatomical ROIs between bFFE CS1.3 and other sequences. TSE produced larger conductivity values than bFFE CS1.3, and bFFE CS4 up to 12 produced smaller conductivity values than bFFE CS1.3. bFFE CS4 and CS8 had small mean differences with bFFE CS1.3. Conductivity values from bFFE CS4 up to 8 had narrow limits of agreement, while limits of agreement of TSE and bFFE with CS factors > 8 were much wider.

The ultra-fast bFFE with dynamic 1.89 s also showed good precision with coefficient of variances over the 520-point time course of 6.67% for GM and 7.83% for WM.

### The effect of RF shimming calibrated with different ${{\varvec{B}}}_{1}^{+}$ mapping methods

**Fig. 5:**
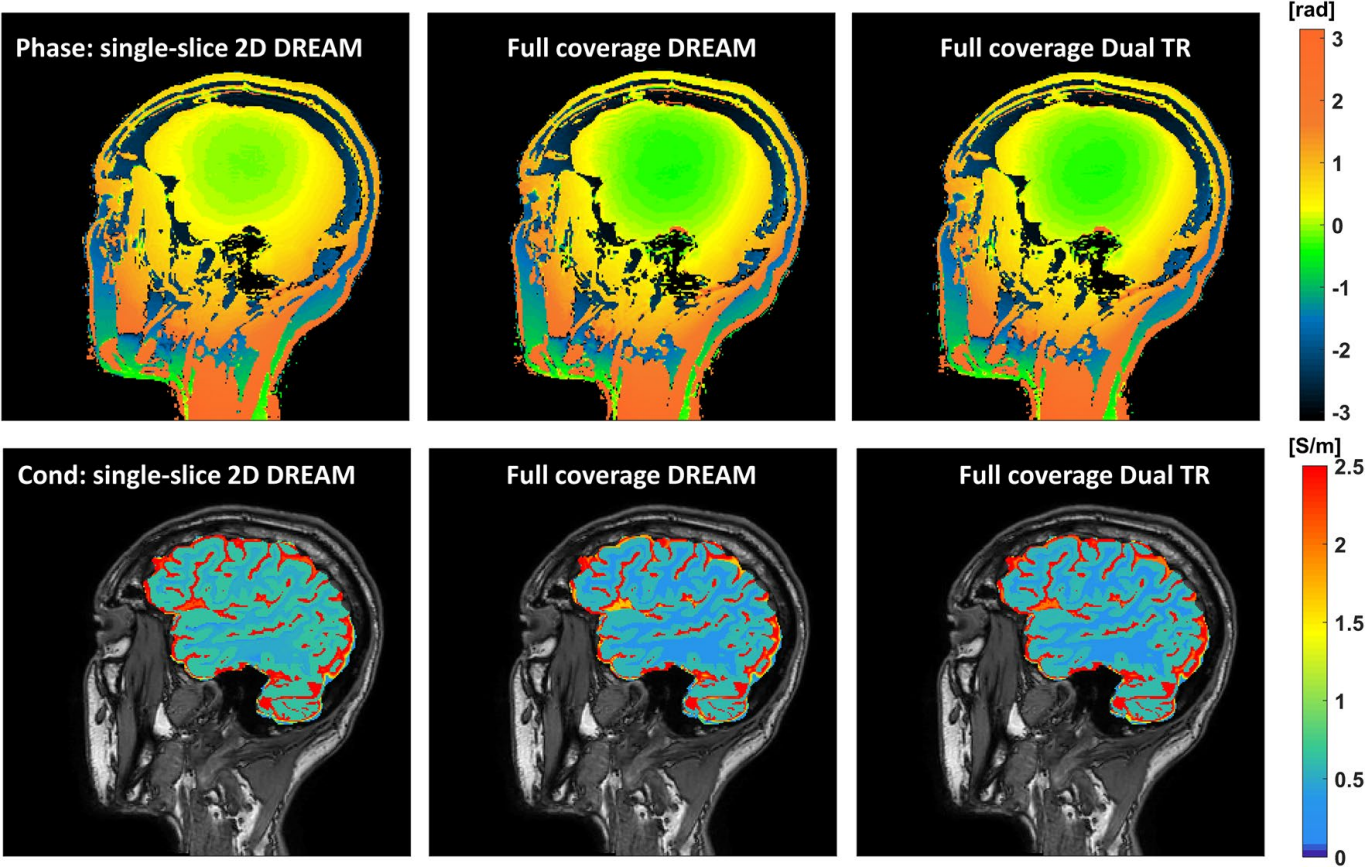
Phase and conductivity maps of one sagittal slice of the same healthy subject scanned with bFFE using 2D DREAM, bFFE using full coverage DREAM and bFFE using full coverage dual TR approach, respectively

**Fig. 6:**
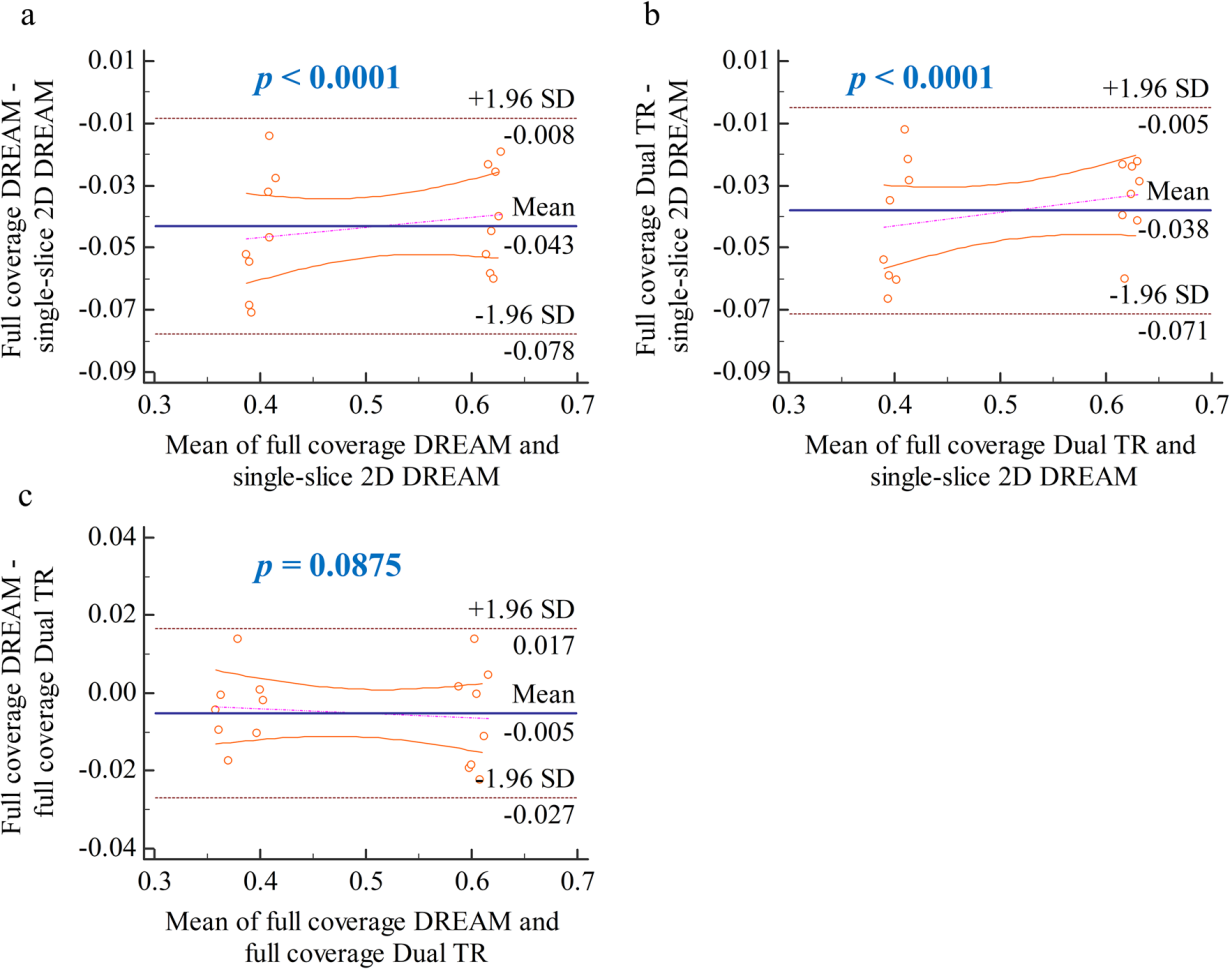
Bland–Altman plots of conductivity measurements and *p*-values of paired t-test (α = 0.05). **a** bFFE using single-slice 2D DREAM and bFFE using full coverage DREAM; **b** bFFE using single-slice 2D DREAM and bFFE using full coverage dual TR approach; and **c** bFFE using full coverage DREAM and bFFE using full coverage dual TR approach

Figure 5 shows phase and conductivity maps of the same sagittal slice from one subject. Both full coverage 2D DREAM and full coverage 3D dual TR approach enlarged the regions of good phase coherence, making the conductivity values much closer to the reference as shown in Table 4. Figure 6 shows Bland–Altman plots of conductivity measurements between the three ${B}_{1}$ mapping methods, and the corresponding *p*-values. In each plot of Fig. 6, there were two pairs of GM conductivity measurements and two pairs of WM conductivity measurements for each of the four subjects. Compared with single-slice 2D DREAM, the mean differences were -0.043 S/m for full coverage 2D DREAM and − 0.038 S/m for full coverage 3D dual TR approach. There was no significant difference between full coverage 2D DREAM and full coverage 3D dual TR.

**Table 4:** Conductivity measurements from one healthy subject using bFFE with three ${B}_{1}$ mapping methods [unit: S/m]

	Single-slice 2D DREAM (Mean ± SD)	Full coverage 2D DREAM (Mean ± SD)	Full coverage 3D Dual TR (Mean ± SD)	References
GM	0.645 ± 0.033	0.596 ± 0.034	0.603 ± 0.037	0.59 [44]
WM	0.423 ± 0.035	0.361 ± 0.028	0.363 ± 0.030	0.34 [44]
CSF	2.229 ± 0.337	2.182 ± 0.315	2.165 ± 0.428	2.14 [44]

## Discussion

Conductivity measurements from phantom saline solutions using bFFE were not significantly different from the true conductivity values, validating the MR acquisition and conductivity reconstruction approaches used here. Conductivity measurements using TSE and bFFE with CS factors were repeatable but the two methods delivered significantly different conductivity values (Fig. 4b). The mean and variance of the conductivity measurement using TSE phase were larger than those measured by bFFE with bFFE values being closer to the reference values. This is consistent with a previous sequence comparison study where the phantom conductivity reconstructed from spin echo transceiver phase had larger median values than that from bFFE phase [49]. This suggests that, despite TSE delivering more complete phase maps of the brain (Fig. 1), bFFE as deployed here is a more precise and accurate method for measuring brain tissue conductivity.

Conductivity measurements using bFFE showed minimal deviation with CS factors up to 8, with deviation increasing at CS factors > 8. With higher CS factors, subcortical structures were more likely to produce less consistent measurements than cortical parcellations. Depending on the area of the brain being measured, the scan can be safely accelerated with compressed SENSE without sacrifice of precision (Fig. 3), offering the potential to employ MREPT in clinical research and applications.

The development of modern MRI scanner hardware has significantly improved the quality of image phase maps. RF shimming and parallel excitation using independent multi-channel coils mitigate the inhomogeneity of the ${B}_{1}^{+}$ field, which can further support one essential assumption of phase-based MREPT that the local variation of magnitude of ${B}_{1}^{+}$ field is much less than the variation of phase [50]. Fully digital RF receive systems, which digitize the signal in the coil and avoid all intermediate analog stages, facilitate a higher SNR and improve the stability of signal and phase. Conductivity measures made with TSE and bFFE are both very repeatable, and the low variance of the calculated conductivity now allows the potential application of MREPT in clinical disease diagnosis. Furthermore, RF shimming calibrated with ${B}_{1}^{+}$ mapping methods would further reduce the measurement error and facilitate more accurate conductivity reconstruction. For the full slice coverage 2D DREAM and especially 3D dual TR, the increase in accuracy comes from making the sequences non-selective which in general is good for ${B}_{1}$ mapping, and from using the same RF pulse shape as that of the 3D bFFE sequence it is correcting. Precision is also improved since these maps are acquired with the same FOV, slice orientation, and slice angulations as bFFE scan.

Conductivity measurement using bFFE shows smaller variance than with TSE (Fig. 3), possibly due to the higher SNR detected by bFFE. bFFE phase was reported to reach a constant plateau between periodic phase wraps (1/TR) in the presence of a linear gradient, meaning that bFFE image phase is not very sensitive to ${\mathbf{B}}_{0}$ inhomogeneity [25]. Additionally, phase contributions from flow and motion were less obvious in bFFE. These features make bFFE more suitable for conductivity measurement with phase-based MREPT. Reconstructed conductivity of CSF still had a larger variance than that of GM and WM, which was consistent as reported that the CSF conductivity reconstruction was more significantly affected by cardiac pulsation than GM and WM [51]. The reference conductivity values of brain tissues at the 3 T Larmor frequency are 0.34 S/m for white matter, 0.59 S/m for gray matter, and 2.14 S/m for CSF [44, 45, 52]. The conductivity values of brain tissues measured using bFFE in our experiment agreed with these reference values while those found with TSE were significantly different to those obtained with bFFE across many brain regions.

In compressed SENSE acceleration, the images are transformed into a wavelet domain where these data could be sparsely represented and the noise could be separated. Pseudorandom under-sampling is performed resulting in variable sampling density in *k*-space where the center is more densely sampled. Thus, increased CS factors shorten the scan duration and increase the denoising level in the wavelet domain. Larger CS factors (> 8) resulted in excessive denoising, severely degraded the quality of bFFE phase and led to underestimated conductivity values. Here, conductivity variance increased with increasing compressed SENSE factor as might be expected due to the consequently reduced resolution and SNR. Compressed SENSE can be used to accelerate bFFE scans reliably up to a factor of 8 (Fig. 3, Fig. 4, Table 2) although lower factors should be used if trying to measure conductivity reliably from small volumes of tissue. Baseline maps with conductivity variance of < 5% could be safely accelerated with compressed SENSE 6 without sacrifice of precision. Precision could be further improved by whole head ${B}_{1}$ mapping, e.g. using full coverage DREAM (Table 4) although this would incur a time penalty.

A precise and repeatable imaging modality is important for clinical applications, not just for reliability of diagnostics but also because the quantitative measurement of conductivity could be used in the diagnosis of tissue abnormalities. For example, if benchmark conductivity values in tumors are obtained from large-scale patient groups, a conductivity map from a patient could provide useful information for diagnosis and monitoring. A fast acquisition time improves the acceptability of the scan for patients, and adds to the flexibility of practice schedule, while still achieving precise measurement.

## Conclusion

bFFE can be safely accelerated with compressed SENSE without sacrifice of precision. Better field mapping further improves the precision of the conductivity measures. BFFE with compressed SENSE offers the potential of MREPT in clinical applications, providing fast, repeatable tissue electrical properties for disease diagnosis and monitoring.

## Data Availability

Data from this study is available at 10.26190/unsworks/24174
